# The tendency to dehumanize, group malleability beliefs, and perceived threat from migrants in Hungary

**DOI:** 10.3389/fpsyg.2022.910848

**Published:** 2022-11-10

**Authors:** Benedek Paskuj, Gábor Orosz

**Affiliations:** ^1^Department of Psychology and Human Development, Institute of Education, University College London, London, United Kingdom; ^2^Université d’Artois, Unité de Recherche Pluridisciplinaire Sport Santé Société, Sherpas, Liévin, France

**Keywords:** dehumanization, group malleability, threat, Hungary, migrant, refugees, essentialism, prejudice reduction

## Abstract

Examining the humanness attributed to several groups in a comprehensive Hungarian sample (*N* = 505) at the height of the “European refugee crisis of 2015,” we found that Hungarians dehumanize Eastern ethnic groups more and Western ethnic groups less than they do to their own ethnic ingroup. Interestingly, we also found that a general tendency of dehumanization is expressed across all national groups. This general tendency of dehumanization was strongly associated with threat perceived from migrants, but the relationship was mediated by group malleability—the belief that human groups can change and are not set in their ways irreversibly. Malleability beliefs were negatively linked to dehumanization tendencies and threat perceived from migrants. We discuss the theoretical and practical implications of the findings that point to the critical role of fixed mindsets about groups in the mechanisms linked to prejudice in a highly xenophobic Hungarian context.

## Introduction

*“Meanwhile, all security experts in the world have already told that there are obviously IS terrorists in the hordes swarming into Europe. Because even if they are animals, they are not stupid.”*

Zsolt Bayer (founding member of Hungarian populist ruling party, Fidesz, on refugees).^1^

### Sociopolitical background and the perception of asylum seekers in Hungary

In the course of 2015, over a million asylum seekers, many of them fleeing from the Syrian civil war, arrived at the borders of the European Union, tipping it into a humanitarian crisis—arguably the continent’s worst since World War II. The reception was mixed. In Sweden or Germany, they found a predominantly warm welcome, but as the migration wave intensified the expression of hostile attitudes, both in the press and in the highest political circles, became increasingly common. Denmark passed a law allowing the confiscation of asylum seekers’ valuables to finance their welfare provision and Hungary, one of the countries along the main overland migration route, erected a 4-m-high fence on its southern border, sealing off the country to unmonitored border-crossings. Launched with a national anti-immigrant billboard campaign in this period, the government subsequently made xenophobic propaganda the central message of its political communication. Building on the Hungarian population’s exceptionally xenophobic sentiments [Nyíri, 2003; European Social Survey European Research Infrastructure (ESS ERIC), 2022], in the years following, they linked everything, from high culture to the coronavirus, to the threat of immigration. Consequently, fear of culturally dissimilar immigrants in the country rose further and the government sailed to sweeping re-elections.

Yet, in 2015, it was not only Hungarian public discourse that took a nefarious turn; degrading voices and dehumanizing rhetoric became prevalent across European media even surfacing in its moderate mainstream (Chouliaraki and Stolic, 2017). The influx of refugees to the EU decreased in the following years, but with the war in Ukraine, it may soon surpass the number of those coming from the Middle East previously. Xenophobic political profiteering across the EU creates tension that the block must address as its population ages and it increasingly relies on immigrants to fill jobs in the care economy and various other sectors.

Understanding the perception of refugees and the potential leverage points for interventions to target can facilitate the successful integration of the newcomers. Our study explored the link between Hungarians’ proclivity to dehumanize and the threat they perceived from migrants, theorizing that thinking of people and groups as malleable entities could attenuate the link between threat and dehumanization.

### Prior research on dehumanization of refugees

Dehumanization, the denial of full humanness, attracted attention in the social sciences after World War II, yet experimental social psychology’s interest has only been drawn to the topic in the past 20 years (Haslam and Loughnan, 2014; Haslam and Stratemeyer, 2016). Seminal work on infrahumanization by Leyens et al. (2000, 2007) focused not so much on genocide or war contexts, but on subtle manifestations of dehumanization between groups that compete within fundamentally peaceful and prosperous conditions (Vaes et al., 2003). Subtle dehumanization has been studied in humanitarian contexts ranging from natural disasters, (Cuddy et al., 2007; Andrighetto et al., 2014) to immigration, where Esses et al. (2013) demonstrated how media portrayals and three tropes—refugees as (1) vectors of infectious diseases, (2) bogus queue-jumpers, and (3) claimants harboring terrorists - contribute to the dehumanization of asylum seekers in the Western world. These three tropes featured heavily in European media during the ‘migrant crisis’, compounded by Islamophobic voices that framed the influx of migrants as a threat to a ‘Christian Europe’. While findings demonstrate the relevance of studying subtle forms of dehumanization, others have recently argued for the need to re-focus attention on its blatant forms, reasoning that these mechanisms are more relevant for hostile behavioral outcomes (Kteily and Bruneau, 2017). With their single-item measurement, the ‘Ascent of Man’, they have presented evidence suggesting that blatant and subtle dehumanization are distinct constructs with differentiable effects (Kteily et al., 2015). They found blatant dehumanization a better predictor of hostile outcomes, such as support for aggressive anti-terrorism policies or retaliatory violence, and more strongly and consistently linked to the support for hierarchy than subtle forms of dehumanization. During the 2015 European ‘migrant crisis’, they found that blatant dehumanization played an important role in the rejection of Muslim refugees throughout the continent over and above political ideology and prejudice (Bruneau et al., 2018).

The present work builds on this theoretical background, adapting it for a Hungarian context, where research demonstrates how dehumanizing rhetoric and migrant-related fearmongering are intertwined in media and public discourse (Bernáth and Messing, 2016)—a link needing no further psychological replication (Bruneau et al., 2018). But as frames and discourse shape thinking and sentiment above and beyond their narrower focus (Price et al., 1997; Moskowitz, 2005), in this case the single target group on which it centers, we theorized that due to the all-permeating nature of xenophobic reporting and communications (Kalmar, 2020) in Hungary, a more general tendency of dehumanization (affecting humanity perceptions regardless of group membership) may emerge. Conceptualized on a general level we still expected it to be strongly associated with perceived threat from migrants (both driven by the same media coverage and discourse), but hoped to find a mediator of this link, which could provide insights for prejudice-reduction efforts.

### Implicit theories of personality and the malleability of groups

Research on lay or implicit theories of personality distinguishes fixed (entity) and dynamic (incremental) mindsets: the former holds that personal characteristics are fixed entities, even if one strives to change them, while the latter refers to the belief that personal characteristics are malleable and can change with time or conscious effort (Levy et al., 2001). Pioneered in educational research, implicit theories about human nature have also proved relevant in the domain of group perception. Levy et al. (1998) found that people with fixed mindsets are more likely to endorse, regardless of their valence, societal stereotypes; are more likely to hold negative views about outgroups in times of intergroup conflict; to perceive greater homogeneity within the outgroup; and consequently apply stereotypes more indiscriminately to outgroup members. For entity theorists, traits are a key tool for understanding groups—they do not only see less difference between members of one group but perceive bigger differences between members of different groups. Accordingly, entity theorists are more likely to discriminate against outgroups by recommending harsher punishments for the same crime.

These findings suggest that in the context of intergroup conflict and contact, fixed mindsets foster selective information processing that confirm pre-existing stereotypes, which often leads to entrenched negative views undermining intergroup contact and increasing hostility. Halperin and colleagues have presented corresponding results in the context of protracted intergroup conflict (Halperin et al., 2011, 2012; Goldenberg et al., 2017, 2018). They found beliefs in group malleability a major facilitator of motivations to make intergroup contact and have also demonstrated that intergroup contact was only fruitful when coupled with beliefs about the malleability of the outgroup. Increasing group malleability perceptions led to lower levels of intergroup anxiety and higher motivation to interact with members of the outgroup. In a different post-conflict setting, Bruneau et al. (2022) found group malleability beliefs the most potent mediator (over and above affective pathways like increased empathy or reduced prejudice) between a media intervention that promoted support for peace and the humanization of ex-FARC combatants in Colombia.

While social theory has long (Pico Della Mirandola, 2012) seen the uniqueness of the human species in their ability to change and transform themselves, dehumanization research has only recently started to incorporate the concept of malleability (Bruneau et al., 2022; Landry et al., 2022). In a context where government communications draw on essentializing tropes that emphasize the difference between Hungarians and migrants (laying the foundation for more derogatory stereotyping of the latter), we hypothesized that one’s willingness to reject essentialism and believe that groups can change their ways would mediate the link between perceptions of humanity and threat from migrants. Perceiving migrants as threatening could be coupled with lower belief in people’s ability to change and could lead to more readiness to dehumanize people. Conversely if people see more animalistic characteristics in various in- and outgroups, they may also see humans less capable of changing or learning, which can, in turn, further fuel fear and threat perceptions from migrants who are depicted in dehumanizing and fearmongering ways in Hungarian media. Hungarians lack contact with migrants and actionable interventions need to find levers to reduce threat perceptions and dehumanization tendencies – we hoped to identify one in malleability beliefs.

### Overview of the present research

First, we assess the self-reported blatant dehumanization of different national groups living in Hungary to understand their relationship. Perceptions of global developmental hierarchies are consistent across countries worldwide (Thornton et al., 2012) and Hungary fits this trend, whereby Western European nations are seen as more sophisticated, compared with poorer Eastern European and Asian nations.^2^ Building on these lay perceptions, target groups were selected based on the combination of Human Development Index, GDP/capita, and geographical position relative to Hungary. We selected Germany, Denmark, and the United States as higher and Romania, Turkey, and Bulgaria as lower status countries. Curious if this West–East slope would materialize in the perception of the ingroup, we included three subsets of the Hungarian populace: those who migrated to Western Europe, those who live in Hungary, and those who live in neighboring countries.^3^ Besides a West–East slope in the dehumanization of groups, we expected that group-specific expressions of dehumanization would cluster by geographic region.

After examining the factor structure of dehumanization, we turn to the relationship between this construct, malleability, and threat perceived from migrants. Xenophobia levels are generally higher in Eastern Europe than in the rest of the continent (Doebler, 2014), but even within the region Hungary has been becoming a negative outlier since the 1990s [European Social Survey European Research Infrastructure (ESS ERIC), 2022]. Before migration surged in 2015, xenophobia predominantly manifested in antisemitism and antigypsyism, rather than hostility toward immigrants and Muslims, but following the government-led anti-immigration propaganda the rejection of, and threat perceived from refugees and Islam also climbed to the highest among EU countries [European Social Survey European Research Infrastructure (ESS ERIC), 2022].

In the Hungarian social, political, and media context, a strong association between the dehumanization of Eastern groups and threat perceived from migrants could almost be taken for granted. We were interested in exploring if amid discourse so laden with dehumanizing rhetoric we could instead find a more general readiness to see others as less than human, regardless of group membership. If such a general tendency of dehumanization emerged, we expected it to be linked to higher threat perceived from migrants—those who were more concerned about migrants would also be more ready to dehumanize. With an eye on future interventions, we were also hoping to establish a mediator that could be the basis of efforts to attenuate the link between threat and general dehumanization, or indeed the reduction of either. Malleability perceptions lent themselves for this mediating role as a negative relationship could be expected with both threat and dehumanization—the less one believes that individuals and groups can change the more likely they would be to dehumanize them and the more readily they would perceive migrants as a threat too. In the present study, we sought to establish a relationship pattern, on which experimental work could build in the future.

## Materials and methods

### Participants and procedure

This study employed a comprehensive probability sample of Hungarians who used the Internet at least once a week (see Supplementary materials for details). The present research was conducted with the approval of the Institutional Review Board of the related university and following the Declaration of Helsinki. Participants received detailed information about the aims of the research project, they were assured of anonymity and provided informed consent.

The final sample of 505 Hungarian respondents who gave valid answers was nationally representative, among those who use the Internet at least once a week, for gender (female = 258; 51%), age (*M_age_* = 40.19 years; *SD_age_* = 11.79 years; range 18–60 years), educational attainment (18% primary, 33% secondary, and 49% tertiary), and place of residence (18.6% capital city, 21.4% county capitals, 31.5% towns, 28.5% in villages).

### Measures

#### Blatant dehumanization

**Figure 1:**
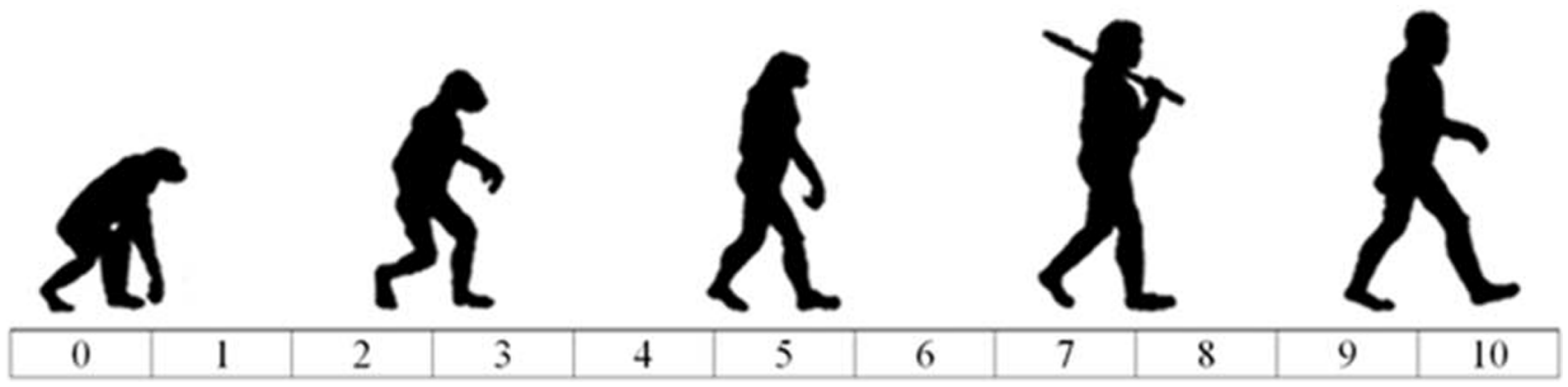
The adapted version of Blatant Dehumanization (Ascent of Man). Instruction: “People can vary in how human-like they seem. Some people seem highly evolved, whereas others seem no different than lower animals. Using the image below as a guide, indicate using the sliders how evolved you consider the average member of each group to be.”

We used the adapted version of the Ascent of Man (Kteily et al., 2015) to capture blatant dehumanization of different groups (e.g., Bulgarians who live in Hungary, Danes who live in Hungary, Hungarians) to assess the intercorrelation between the blatant dehumanization of different social groups. Respondents rated how evolved each group was on an 11-point (ranged between 0 and 10) scale presented below in Figure 1. For the path model, we reversed the scale for higher scores to correspond to more dehumanization.

#### Perceived threat

We adapted and complemented Stephan et al. (2002) threat scale using Beaton et al. (2000) protocol. Seven items (α = 0.96), both realistic and symbolic, measured the individuals’ level of perceived threat from migrants (e.g., *“Migrants represent health risk to Hungarians”*). Respondents indicated their level of agreement using a five-point scale ranging from 1 (Strongly disagree) to 5 (Strongly agree), with higher scores indicating higher levels of perceived threat.

**Groups’ malleability beliefs** were measured with four items (α = 0.95; e.g., *“As much as I hate to admit it, you cannot teach an old dog new tricks* – *groups cannot really change their basic characteristics.”*) rated on a scale from 1 = strongly disagree to 6 = strongly agree (Levy et al., 1998; Halperin et al., 2011). For the model, we computed the composite so that lower scores indicate more fixed, whereas higher scores represent more incremental mindsets.

### Statistical analyses

**Figure 2:**
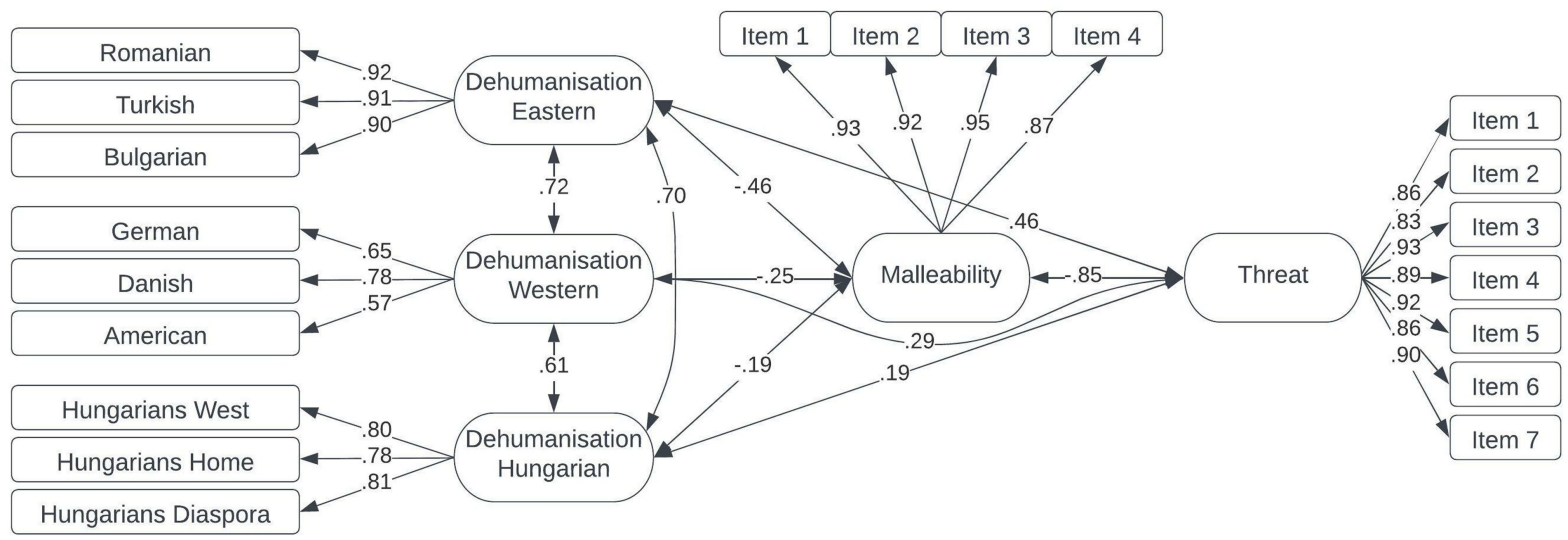
The results of structural equation modeling. Higher dehumanization scores indicate stronger dehumanization, higher malleability scores indicate more malleability and higher threat scores mean stronger threat. Single-headed arrows represent standardized regression coefficients and double-headed arrows represent covariances. All pathways were significant at *p* < 0.001.

Descriptive statistics were produced in SPSS 22, the latent-variable path model was built in Amos 21 using maximum-likelihood estimator (See Figure 2). Multiple goodness-of-fit indices were used (Browne and Shapiro, 2015, see Supplementary materials). For internal consistency, Cronbach’s alpha values were estimated and observed following the guidelines of Nunally and Bernstein (1978) regarding the acceptability of the value (0.70 acceptable, 0.80 good).

## Results

Descriptive data for the dehumanization of different national groups showed a West–East slope (see Supplementary materials; Table 1); Germans were rated as the most evolved followed by Danes and Americans, whereas Turks were rated as the least evolved followed by Romanians and Bulgarians, with Hungarians sitting in between the Western and Eastern outgroups. This West–East slope was expressed even within the ingroup, but these differences did not reach statistical significance.

**Table 1:** Descriptive statistics and intercorrelations between the prejudice variables.

	Range	Mean	SD	1	2	3	4
1. Dehumanization Western	0–10	9.19	1.13	_			
2. Dehumanization Hungarian	0–10	8.46	1.78	0.465^**^	_		
3. Dehumanization Eastern	0–10	7.48	2.34	0.583^**^	0.608^**^	_	
4. Malleability	1–5	4.25	1.52	−0.207^**^	−0.158^**^	−0.399^**^	_
5. Perceived threat	1–5	3.33	1.37	0.254^**^	0.168^**^	0.418^**^	−0.808^**^

SD, standard deviation; all correlations were significant at **p < 0.001.

According to a simple and parsimonious first-order confirmatory factor analysis (see loadings and covariances in Supplementary materials; Figure 1, CFI = 0.986; TLI = 0.979; RMSEA = 0.056 [90%CI 0.027–0.083]), three factors can be distinguished referring to Eastern dehumanization (Romanian, Bulgarian, and Turkish), Western dehumanization (Danish, German, and United States), and Hungarian dehumanization factors (Western diaspora, Eastern diaspora, and Hungarians in Hungary). Next, we investigated how the three factors (Eastern, Western, and Hungarian) of dehumanization are related to threat and how the links are mediated by group malleability (for descriptives, see Table 1 and for the path model see Figure 2).

Without assumptions about the direction of causality, we calculated covariances between the latent variables. Fit indices were acceptable (CFI = 0.970; TLI = 0.965; RMSEA = 0.059 [90%CI 0.053–0.066]). Dehumanization of Eastern groups had a moderate and negative association with group malleability (*β* = −0.46, *p* < 0.001), which, in turn, was strongly negatively linked with perceived threat from migrants (*β* = −0.85, *p* < 0.001). Furthermore, dehumanization of Eastern groups was positively related to threat (*β* = 0.46, *p* < 0.001). We can see a similar relationship pattern between Western and Hungarian dehumanization and group malleability (*β* = −0.25, *p* < 0.001, *β* = −0.19, *p* < 0.001) as well as threat (*β* = 0.29, *p* < 0.001, *β* = 0.19, *p* < 0.001) with somewhat weaker relationships. The link between the three dehumanization factors and threat from migrants is weaker compared with the link between malleability and threat. We found that group malleability perceptions mediated the link between dehumanization of various groups and perceived threat from migrants.^4^

## Discussion

In our study, we set out to assess the blatant dehumanization of different national groups and how tendencies to dehumanize are linked to perceiving migrants as a threat and incremental mindsets about human groups. We hoped that malleability beliefs could disrupt the self-perpetuating cycle between threat perceived from migrants and general dehumanization tendencies—that belief in the ability of human groups for change could be a lever for interventions looking to alleviate xenophobia in a challenging Hungarian context—after proving its utility in basic (Landry et al., 2022) and applied (Bruneau et al., 2022) science.

We replicated the mental slope of evolvedness that had been established in previous Hungarian studies: participants rated Western nations as the most evolved, followed by Hungarians, and rated the Eastern (relative to Hungary) nations as the least evolved (Melegh, 2006). We then went on to assess how attributing humanity to different target groups is associated with perceived threat from migrants and implicit theories about the malleability of a group and its members. Based on previous research on intergroup conflict (Halperin et al., 2011; Weiss-Klayman et al., 2020), we expected that the link between the dehumanization of others and perceived threat would be mediated by general perceptions of group malleability and this we indeed found. Our model revealed that the negative relationship between the tendency to dehumanize and perceived threat was partially mediated by group malleability perceptions. One interpretation is that the more one saw the humanness of other people in general, the more likely they were to see people as malleable, and the less threat they perceived from migrants. Conversely, the more one saw other people as animals, the less likely they were to see people as malleable and the more threat they perceived. Another interpretation is that perceived threat can lead to dehumanization through the belief that groups cannot change—for those who hold incremental views about social groups threat leads a lot less to dehumanization. From this perspective, migrant threat could decrease group malleability perceptions which then contributes to dehumanization.

### Theoretical implications

Proliferating dehumanization research in the past two decades has identified individual differences and situational factors that make people more likely to mentally strip others of their full humanity. Studies have established social dominance orientation as a reliable individual difference variable predicting differences in the dehumanization of outgroups (Haslam and Loughnan, 2014), whereas a more affective approach showed that disgust-sensitivity is one non-attitudinal trait enhancing the likelihood of outgroup dehumanization (Hodson and Costello, 2007; Buckels and Trapnell, 2013). More contextual contributing factors of dehumanization include power (Lammers and Stapel, 2011), threat (Goldenberg et al., 2009; Kteily et al., 2016), and motives to enhance the ingroup (Castano and Giner-Sorolla, 2006).

Our high intercorrelations between the perceptions of several groups’ humanity are strongly indicative of a tightly knit mental web where a forceful impact on the perception of a single group’s humanness will not be compartmentalized but affect the dehumanization of other groups too. Research on dehumanized perception argues that perceiving the mind of others is an effortful social cognitive mechanism that may or may not be spontaneously engaged, depending on a host of situational variables (Harris and Fiske, 2011). Prior research investigated the evaluation of several target groups by the same respondents (Kteily et al., 2015); however, our results are the first to establish that in Hungarian peoples’ minds the perception of one group’s humanness is closely linked to that of other groups and the tendency to dehumanize is expressed consistently across the explicit ratings of different target groups on the Ascent of Man scale. While only further research can establish whether this is a consistent individual difference, independent from specific group membership, we believe that considering the individual’s tendency to de/humanize and not only conceptualizing dehumanization as a difference score between in- and outgroup perceptions can extend our understanding of intergroup relations, especially in contexts where public discourse is increasingly polarized (US) or where control over media is used to fuel dehumanizing rhetoric (in several hybrid regimes).

With flare-ups of antigypsyism and homophobia in recent years, it is our concern that hostile and dehumanizing discourse of one vulnerable group in Hungary (in this case refugees) can facilitate the expression of hostile attitudes toward other vulnerable groups, especially when aided by derogatory media coverage. As long as group-based perceptions of humanity remain tightly connected, attitude change toward a single outgroup (like Muslim refugees) could also face obstacles. If one is motivated to enhance the ingroup, by maintaining the ‘humanness gap’ to the outgroups, the limit to the ingroup’s further humanization can determine the extent to which perceptions of other groups can improve.

### Applied implications

Behavioral and communication interventions need to find the opening in the process of attitude formation to successfully address fearful and hostile feelings toward immigrants. The idea that humans are the only species capable of transforming themselves has been present in intellectual history since at least the renaissance, so it should come as no surprise that incremental views about social groups have a significant ability to neutralize the link between perceived threat and the animalistic representation of others. This result dovetails with findings on how the promotion of incremental mindset about groups and, in particular, the threatening outgroup, is a most promising avenue toward fostering dialog and tolerance (Halperin et al., 2011, 2012). This dialog and the majority’s motivation to connect will be key for the hopes of peacefully and successfully integrating newly arrived refugees in Europe.

Though the discourse around immigration took a hostile turn during the ‘European migrant crisis of 2015’, Hungary is still an outlier in Europe in that its government spends vast sums on xenophobic campaigns. While research from elsewhere (Prati et al., 2015) offers counter-stereotypes as a lever, very few prejudice-reduction interventions are effective in the Hungarian context (Orosz et al., 2016). Based on our findings future experimental studies seeking to decrease dehumanization should strongly consider the manipulation of malleability perceptions. Perceived threat is actively stoked by government communications in the country and group malleability could be one of the few keys to minimizing perceived threat’s automatic translation into more general outgroup derogation. Finally, our results suggest that practitioners working on the integration of newcomers would benefit from thinking about perceived threat, dehumanization, and malleability perceptions in host communities (cf. McLoughlin and Over, 2019).

### Limitations

Though we had a comprehensive sample, representative in many respects, the over-representation of participants with higher levels of education could be perceived a limitation of our sampling method that collected data from those who use the internet at least weekly. Additionally, although the Ascent of Man measure has excellent psychometric properties, all measures were self-report scales, and future research exploring the relationship between dehumanization, malleability, and perceived threat should consider the usage of implicit or behavioral measures that are less susceptible to respondent bias. Perhaps in less galvanized European countries, dehumanized perceptions of specific target groups are more segmented and do not covary with the dehumanized perceptions of other groups. To find out, research should replicate our results across countries, with relevant target groups. Finally, we intentionally used covariances in the model, as our correlational design does not allow for causal inference (see Rohrer et al., 2022 for more on the limitations of path models). Future intervention studies building on our findings will need to employ experimental designs to verify causal directions between the investigated concepts.

## Data availability statement

The raw data supporting the conclusions of this article will be made available by the authors, without undue reservation.

## Ethics statement

The studies involving human participants were reviewed and approved by Eotvos Lorand University, Kutatasetikai Bizottsag (KEB). Written informed consent for participation was not required for this study in accordance with the national legislation and the institutional requirements.

## Author contributions

BP and GO contributed to the study design, literature review, data gathering, manuscript writing, data analysis, and interpretation. All authors commented on the draft and contributed to the final version, approved the publication of the manuscript, and agreed to be accountable for all aspects of the work.

## Funding

The last author was supported by the Young Researcher STARS grant from Conseil Régional Hauts de France and by the Strategic Dialogue and Management Scholarship (Phase 1 and 2).

## Conflict of interest

The authors declare that the research was conducted in the absence of any commercial or financial relationships that could be construed as a potential conflict of interest.

## Publisher’s note

All claims expressed in this article are solely those of the authors and do not necessarily represent those of their affiliated organizations, or those of the publisher, the editors and the reviewers. Any product that may be evaluated in this article, or claim that may be made by its manufacturer, is not guaranteed or endorsed by the publisher.

## Footnotes

^1^ https://www.magyarhirlap.hu/velemeny/Elkerulhetetlen

^2^Findings on the link between dehumanization and the social status of the target group are mixed – while there are studies to suggest (Lammers and Stapel, 2011; Capozza et al., 2012; Fourie et al., 2022) that the low status of a target group predicts its dehumanization, others (Rodríguez-Pérez et al., 2011) found no such relationship across perceptions of various national groups globally. This could be explained by the differential dehumanization tendencies associated with low and high-status groups (Sainz et al., 2019).

^3^After WWI Hungary lost significant part of its territory, and up until today there is a diaspora of about 1.5 million Hungarians who preserved their language and culture in neighbouring countries.

^4^Moderation analyses demonstrated that interaction between dehumanization and malleability was not significant on threat (*p* = 0.93). This was not only the case for overall dehumanization, but for the Eastern (*p* = 0.79), Western (*p* = 0.48) and Hungarian (*p* = 0.83) groups’ interaction with malleability.
