# Supplementary material for: The tendency to dehumanize, group malleability beliefs, and perceived threat from migrants in Hungary
Source: Front Psychol. 2022 Nov 10;13:910848. doi: 10.3389/fpsyg.2022.910848 (PMC9685616; doi:10.3389/fpsyg.2022.910848)
Supplement: Supplementary file 2 [file Table_2.DOCX]

**Supplementary Materials**

**Sampling details:**

Participants were selected randomly from 15,000 panel members with the help of a market research company (Solid Data) in June 2016. For data collection, a multiple-step, proportionally stratified, probabilistic sampling method was employed, in which individuals were removed from the panel if they gave responses too quickly and/or had fake e-mail addresses.

|  |  | Sample | 2016 census |
| --- | --- | --- | --- |
| Gender | Male | 49% | 48% |
|  | Female | 51% | 52% |
| Age | Mean | 40.2 | 42.4 |
| Education | Primary | 18% | 27% |
|  | Secondary | 33% | 54% |
|  | Tertiary | 49% | 19% |
| Place of residence | Capital | 18.6% | 17.9% |
|  | Towns | 52.9% | 52.7% |
|  | Villages | 28.5% | 29.4% |

**Goodness of fit indices considered for the latent-variable path model:**

Comparative Fit Index (≥0.95 for good, ≥0.90 for acceptable), Tucker-Lewis Index (≥0.95 for good, ≥0.90 for acceptable), the Root-Mean-Square Error of Approximation (≤0.06 for good, ≤0.08 for acceptable), and Standardized Root Mean Square Residuals (≤0.05 for good, ≤0.10 for acceptable).

**Table 1.** *Dehumanization of different groups*

|  | Range | Mean | SE | SD |
| --- | --- | --- | --- | --- |
| Germans | 0-10 | 9.35 | 0.05 | 1.23 |
| Danes | 0-10 | 9.14 | 0.07 | 1.52 |
| Americans (US) | 0-10 | 9.08 | 0.07 | 1.54 |
| Hungarians migrated West | 0-10 | 8.54 | 0.09 | 1.94 |
| Hungarians in Hungary | 0-10 | 8.45 | 0.10 | 2.15 |
| Hungarians in surrounding countries | 0-10 | 8.40 | 0.09 | 2.05 |
| Bulgarians | 0-10 | 7.70 | 0.10 | 2.32 |
| Romanians | 0-10 | 7.14 | 0.12 | 2.78 |
| Turkish | 0-10 | 7.04 | 0.12 | 2.70 |
| Syrians | 0-10 | 6.35 | 0.15 | 3.26 |

*Note.* SE = standard error; SD = standard deviation

**Figure 1.** *Confirmatory Factor Analysis*

**
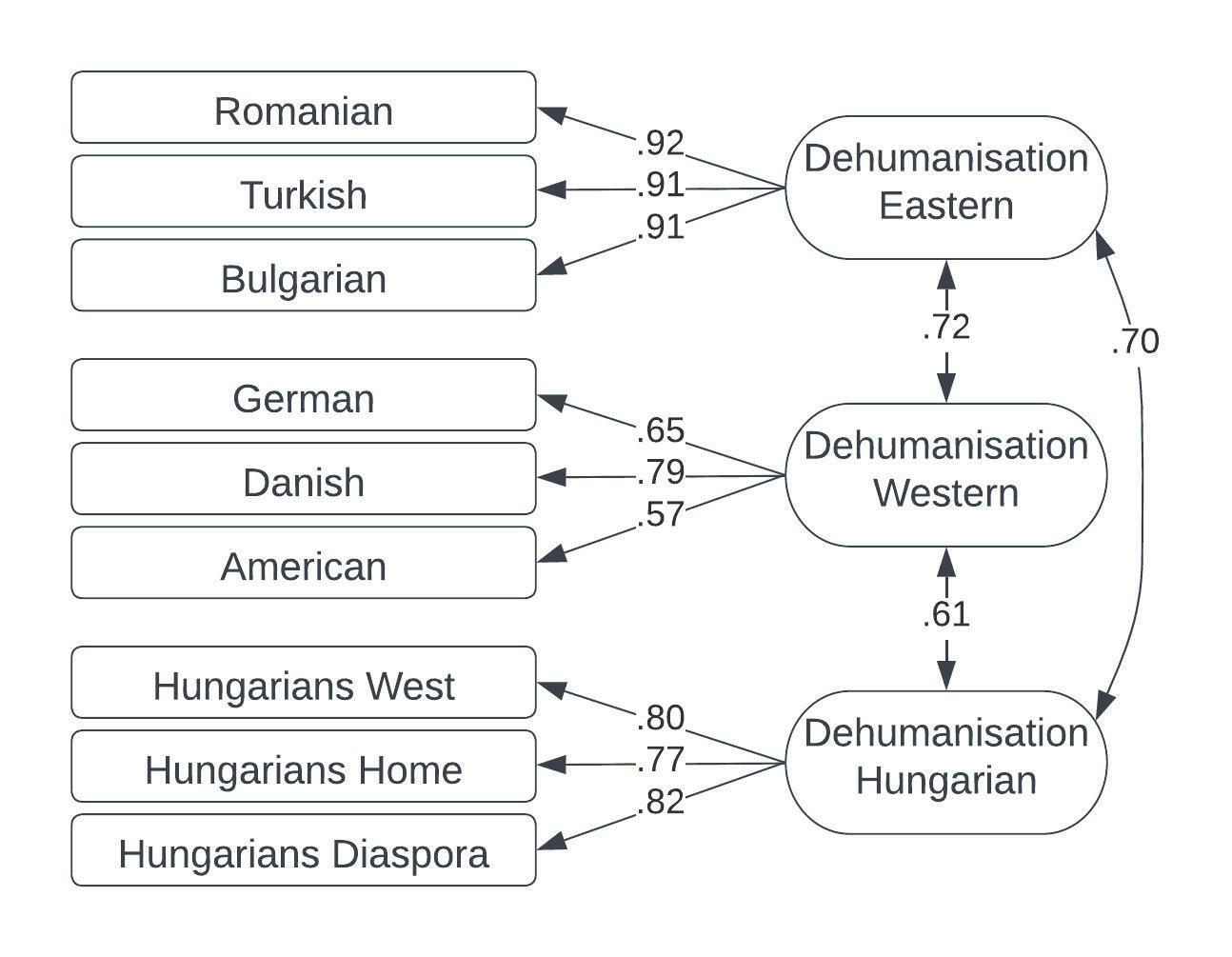
**

*Note.* Single-headed arrows represent standardized regression coefficients and double headed arrows represent covariances. All pathways were significant at p < .001.
